# 0920. Cerebrovascular autoregulation in normotensive hypovolaemia: a study combining lower body negative pressure and MRI

**DOI:** 10.1186/2197-425X-2-S1-O28

**Published:** 2014-09-26

**Authors:** SC Beards, S Brodie, J Cain, LM Parkes, A Jackson

**Affiliations:** University Hospital of South Manchester, Acute Intensive Care Unit, Manchester, UK; Manchester University, Wolfson Molecular Imaging Centre, Manchester, UK

## Introduction

Previous studies using transcranial Doppler sonography have suggested that cerebral circulation is preserved relative to cardiac output during normotensive hypovolaemia (NTH). We have further investigated the cerebral autoregulatory responses to hypovolaemia using using MRI measurements of total cerebral blood flow combined with graded levels of lower body negative pressure (LBNP) to induce steady state central hypovolaemia.

## Methods

11 healthy male volunteers (21-24 years) underwent MRI scanning with their legs and torso within a LBNP chamber; phase contrast angiography based flow measurements (MRI-PCA) were acquired at 10mmHg increments from baseline, to -50 mmHg . Measurements were made through the carotid and vertebral arteries to measure total cerebral volume flow (CBVF) and through the ascending and proximal descending aorta to measure cardiac output (CO).

## Results

**Figure 1:**
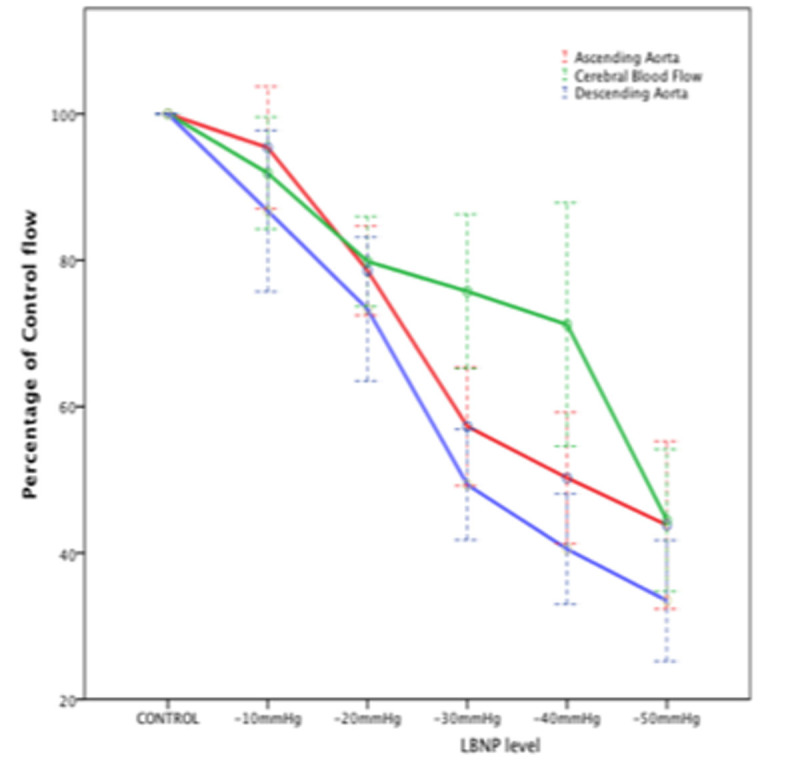
Figure 1

At low levels of LBNP (≤ -20mmHg) decrease in CBVF was directly proportional to the decrease in CO (Figure 1). Between LBNP of -20 mmHg and -40 mmHg there was a relative maintenance of CBVF in relation to cardiac output (P< 0.05) in keeping with classic, pressure mediated, cerebrovascular autoregulation. LBNP of -50mmHg produced a decrease in CBVF of 65.5% decrease (P< 0.01) in keeping with a 63.6% (P< 0.01) decrease in CO.

## Conclusions

These findings demonstrate a correlation between decreases in CO and CBVF during normotensive hypovolaemia (NTH), which is in disagreement with previous studies using transcranial Doppler to measure CBVF. The findings do not support previous descriptions of relative sparing of CBVF due to compensatory reductions in cerebrovascular resistance in response to NTH ^1^. This difference is likely to reflect the different methods of cerebral blood flow measurement. MRI-PCA allows direct volume flow measurements from the carotid and basilar arteries whereas TCD uses flow velocity in the middle cerebral arteries as a surrogate of volume flow. This potentially important discrepancy requires further investigation to elucidate the mechanisms controlling CBF responses to nth.
